# A patient with weakness and an abnormal chest radiograph: A case report

**DOI:** 10.7196/AJTCCM.2018.v24i1.183

**Published:** 2018-04-03

**Authors:** A van Straaten, J A Shaw, C F N Koegelenberg

**Affiliations:** Division of Pulmonology, Department of Medicine, Tygerberg Academic Hospital and Stellenbosch University, Cape Town, South Africa

**Keywords:** mediastinal mass, weakness

## Abstract

A 40-year-old black male presented to ICU after intubation for airway protection due to rapid onset of neck weakness and swallowing difficulty. His chest radiograph showed an unusual mediastinal opacity for which a computer tomography (CT) scan was done, confirming a mediastinal mass.

## Background

Myasthenia gravis (MG) is a disorder of the neuromuscular junction (NMJ) which results in localised or generalised fatigable weakness of skeletal muscles, commonly with ocular involvement.^[1]^ There are paraneoplastic forms (thymoma-associated) and non–paraneoplastic forms, and the disorder is immunologically heterogeneous.^[2]^ Approximately 10% of patients with MG have a thymoma, while up to a third of patients with a thymoma will develop MG. Pyridostigmine is the preferred symptomatic treatment, and if patients do not adequately respond to this therapy, corticosteroids, azathioprine and thymectomy are first-line immunosuppressive treatments. There are further immunomodulatory drugs emerging, but they are limited by the scarcity of controlled studies. Long-term drug treatment is essential for most patients and must be tailored to the particular form of MG.^[3]^ Thymectomy is indicated as first-line therapy in all patients with thymoma or suspected thymoma, regardless of the status of MG.

## Case report

A 40-year-old black male, who was employed as a long-distance truck driver, presented with a history of rapid onset of neck weakness, swallowing difficulty and ptosis. The treating physicians intubated the patient for airway protection, initiated antibiotic treatment for a suspicion of aspiration pneumonia and transferred him to an intensive care unit (ICU).

**Fig. 1:**
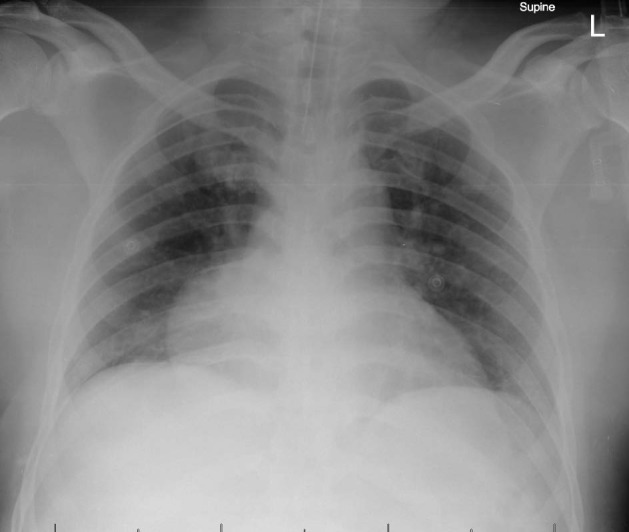
Chest radiograph of patient on arrival in ICU

On arrival in the ICU the patient had a temperature of 38.6 °C without an obvious source of infection. His other vital signs were within normal limits. He was noted to have bilateral ptosis and a bulbar palsy. Power in all limbs was normal. The rest of the examination was unremarkable. A chest radiograph (Fig. 1) was performed on his arrival at the ICU.

**Fig. 2:**
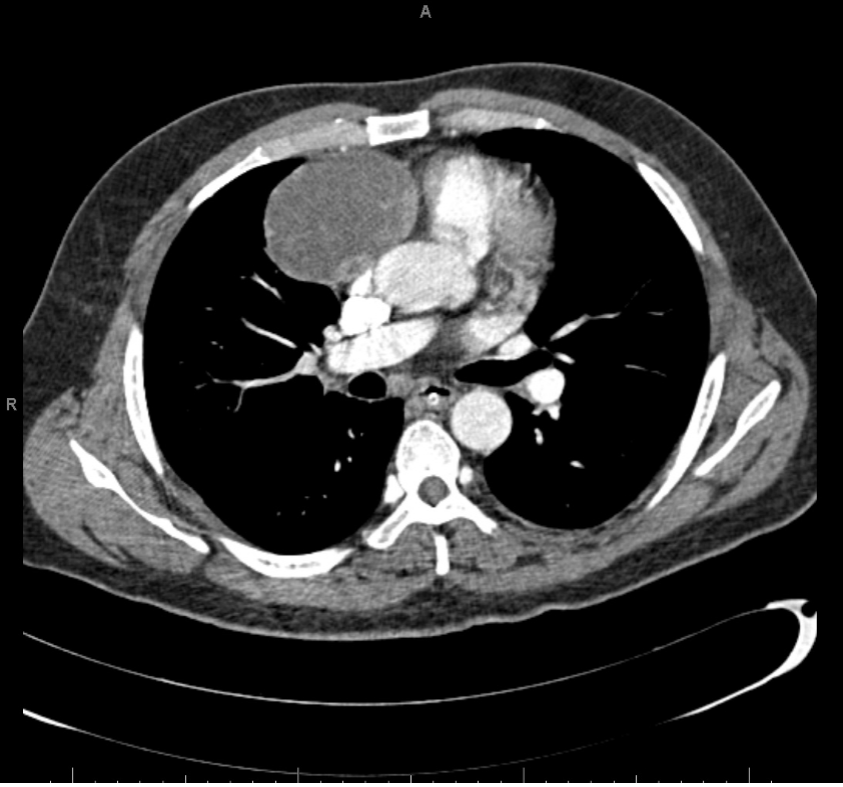
A computed tomography scan of the patient’s chest showing an anterior mediastinal mass.

During his admission to the ICU, a computed tomography (CT) scan of the patient’s chest was performed to explore a radiological abnormality, which revealed a well-circumscribed low-anterior mediastinal mass (Fig. 2).

Anti-acetylcholine receptor (AChR) antibody testing was positive, and nerve conduction studies demonstrated a decremental pattern in keeping with a diagnosis of myasthenia gravis. The patient was treated with plasmapheresis, corticosteroids and neostigmine. He was successfully extubated after 7 days. Subsequent surgical excision of the mediastinal mass confirmed a type B2 thymoma.

## Discussion

### Myasthenia gravis and the pathogenesis of autoimmunity

Myasthenia gravis (MG) is a disorder of the neuromuscular junction (NMJ) which results in localised or generalised fatigable weakness of skeletal muscles, commonly with ocular involvement.^[1]^ While congenital MG results from gene mutations affecting the NMJ components, most patients who develop MG in adulthood have autoantibodies specific to the postsynaptic AChR or functionally related molecules.^[4]^ These autoantibodies are thought to originate in hyperplastic germinal centres in the thymus where myoid cells expressing AChR are clustered.

Autoantibodies are present in the serum in 80% to 90% of cases, most commonly anti-AChR antibodies.^[5]^ However, there is significant immunological heterogeneity, with variation in antibody structure between individuals as well as between different muscles within a single individual. Thymic abnormalities are present in the majority of AChR antibody positive cases, with 60% - 70% of cases having thymic hyperplasia and 10% - 12% having a thymoma.

The thymus contains a small number of myoid cells that are the only known cells to express intact AChR outside of muscle.^[6]^ Thymic epithelial cells produce unfolded AChR subunits that are believed to prime helper T cells to autoimmunity. These T cells then attack the AChR on the myoid cells, creating infiltrating germinal centres in the hyperplastic thymus and triggering complement activation and deposition. The autoimmunisation is completed as the antibodies in the germinal centres diversify to recognize intact muscle AChR.

### Thymomas and MG

While only approximately 10% of patients with MG will have a thymoma, it is known that up to a third of patients with a thymoma will develop MG.^[7]^ The role of thymoma in autoimmunity is not clear, although it is thought that the histological subtype of thymoma may be important. The development of MG is associated with mixed thymomas, but not with thymomas of the cortical type. Certain antibodies are more commonly associated with the presence of a thymoma.^[2]^ In addition to AChR antibodies, some individuals have muscle autoantibodies directed against titin or the ryanodine receptor as well as other intracellular muscle proteins. Among patients with MG, the presence of anti-titin antibodies is predictive of a thymic epithelial tumour (sensitivity 69% - 80%, specificity 90% - 100%). Anti-low-density lipoprotein receptor-related protein 4 (LRP4) and anti-muscle-specific kinase (MuSK) antibodies are not associated with thymomas.

Thymomas are also associated with an increased risk of developing other autoimmune diseases such as thyroiditis, rheumatoid arthritis and systemic lupus erythematosus.^[8]^

### Thymectomy in MG

Thymectomy is indicated as first-line therapy in all patients with thymoma or suspected thymoma, regardless of the status of MG.^[7]^ If complete resection is not feasible, then a biopsy is required prior to neoadjuvant chemotherapy or radiotherapy, to improve likelihood of surgical resection. Patients with MG and a thymoma generally show significant improvement in disease after thymectomy. Moreover, there is evidence for the benefit of thymectomy over drug therapy alone in patients with generalised MG and AChR antibodies, even in the absence of a thymoma.^[9]^ The thymectomy trial for non-thymomatous myasthenia gravis patients receiving prednisone (MGTX), was a multicentre, assessor-blinded trial run between 2006 and 2012, which enrolled 126 subjects with generalised AChR antibody-associated MG.^[7]^ It demonstrated significantly lower severity of weakness, lower prednisone and immunosuppressive agent requirements, reduced need for hospitalisation for MG exacerbations and a greater proportion of subjects with minimal manifestations at 12 months in the thymectomy group compared with the prednisone-alone group.

Evidence also favours thymectomy in early-onset disease rather than in late-onset disease, as the latter group of patients often have thymic atrophy and derive no benefit from the procedure.^[10]^ A thymectomy should also be considered in children with MG.

Certain populations should not undergo thymectomy based on current evidence. Patients with MG and anti-MuSK or LRP4 antibodies should not be offered thymectomy. In patients with pure ocular MG, there is insufficient evidence that surgery prevents generalisation of results in remission; however, it has been argued that thymectomy should be considered in patients with ocular MG when drug treatment has failed if the patients have AChR antibodies and a risk of generalised disease.

### MG in South Africa

A retrospective observational study published in 2007 demonstrated that the annual incidence of anti-AChR-positive MG in the Cape Town metropole of South Africa is similar to that of developed countries, without significant differences in the incidence rates among the three predominant racial groups.^[11]^ However, a difference was noted in the clinical phenotype between the racial groups in that black patients were more likely to develop treatment-resistant complete ophthalmoplegia and ptosis than white patients (18% v. 2%; *p*=0.041). Despite similar-sized cohorts, white patients were more likely to develop generalised myasthenia poorly responsive to therapy (*p*=0.005) than black patients. There were no significant racial differences in the time between diagnosis to initiation of therapy, or the performance and timing of thymectomy.
